# Mr. Hall, on Vaccination

**Published:** 1805-11-01

**Authors:** R. Hall

**Affiliations:** Clements Inn


### *To the Editors of the Medical and Physical Journal.*

Gentlemen,

The characteristic distinctions of pustular diseases are often so extremely minute, and the modifications of which they are susceptible so anomalous, as not always to be easily ascertained; it cannot, therefore, be deemed wonderful, that medical men should have frequently fallen into mistakes in deciding on the true nature of exanthematous affections. Thus, in particular, it is well known, that so great a similarity frequently prevails between what is usually denominated the chicken-pock and small-pox, that the former of these diseases has not unfrequently been mistaken for the latter, and given occasion to the supposition of individuals having suffered a second attack of the small-pox. Medical records abound with cases of this kind previous to the introduction of vaccine inoculation; and it is not less certain, that since this period they have as frequently either been regarded through ignorance, or misrepresentation and a spirit of opposition, as examples of small-pox subsequent to vaccination.

The doctrine that the constitution is rendered insusceptible of variolous infection from having previously undergone the cow-pox, has lately been much controverted, and various cases have been adduced in support of the contrary opinion. But many of these appear, on a more minute investigation, to have originated in mistakes concerning either the former or latter disease, from the matter employed having undergone decomposition, or from its being of a different nature. While this seems to be the fact, respecting a great majority of the cases in question, there are, it must be confessed, a few others, apparently so well authenticated as to create at least some degree of hesitation, as to the universality of the above position, or whether the rule is so general as has been supposed. Admitting, however, that cases of genuine small-pox have really occurred subsequently to vaccination, we are still unable to perceive, with the adversaries of this practice, how such occurrences can militate, or be construed into a valid argument against its adoption, since a few exceptions can never surely be brought into competition with the vast body of evidence in its favour; and since, if this mode of argumentation were available, objections on similar grounds might be urged equally against variolous inoculation.

Though

Though every unprejudiced observer must, in our opinion, be convinced of the superiority of vaccine over variolous inoculation, we yet think that the friends of the former practice have acted very unjustly in endeavouring to repress all further discussion respecting the laws of the vaccine poison, by vilifying those who have attempted, by experimental investigation, to ascertain the permanent efficacy of cow-pox, and how far we may confide in its anti-variolous powers. Freedom of inquiry, and an unrestrained publication of its results, can never retard the acquisition of knowledge. Surely, if any doctrine be founded in truth, it will securely stand the test, and be more fully confirmed by the closest investigation. If, on the contrary, it be erroneous, no greater benefit can be conferred on mankind than the detection of its failures, and the exposure of its errors. Applying this reasoning to the practice in question, it must be obvious that we are equally indebted to those who furnish us with well attested cases, whether adverse or favourable to vaccination. While, however, we contend on the piesent, as well as on every other occasion, for the most unlimited freedom of discussion, no one can reprobate more than ourselves the conduct of a few over zealous anti-vaccinists, who by partial and distorted statements, depreciate the value and efficacy of this important discovery, and represent it as fraught with the most mischievous consequences; for altho' such attempts can only redound to the discredit of their authors, and must ultimately prove abortive, yet by diffusing unnecessary alarm and dismay among the weak and uninstructed part of the community, they are but too well calculated to impede the progress and universal adoption of this salutary practice.

These reflections were suggested to our mind by an incident, which occurred a short time since in the family of Mr. Ross, Newcastle Court, Strand. An eruption having appeared on two of his children, he and the rest of the family were greatly alarmed, supposing it to be the small-pox, which at the time prevailed in one of the neighbouring houses. On this account I was desired to visit them. One of the children, they informed me, had been vaccinated about a year before this period; the other had not been subjected to that process. In both cases, the eruption was extremely copious, but the pustules were much larger and more confluent in the one which had not undergone vaccination. In the latter also the preceding fever was not only unusually severe, but more protracted than than is common in similar cases; while in the former it was comparatively mild, and of much shorter duration. In both, the pustules so exactly resembled, in form, figure, and other circumstances, those of small-pox, that had we founded our opinion on the external character alone, we should most unquestionably have deemed them both cases of genuine small-pox; but as they neither went through the regular course, nor were attended with any of those symptoms which uniformly accompany violent cases of small-pox, such as increased salivary discharge, swelling of the face, affection of the throat and fauces, &c. we did not hesitate to consider them as cases of confluent varicella; and the event afterwards proved that we were not mistaken in this opinion, for the child which had not been vaccinated, on being afterwards subjected to that process, went through the disease in so regular a manner as perfectly to satisfy us, had any doubts existed, on the subject.

Fully aware that the above cases are destitute of novelty, our only motive for transmitting them to the Editors of the Medical and Physical Journal, is a conviction that examples of this kind cannot be too frequently held up, particularly at the present moment, to public view, in order to operate as a caution to practitioners against forming too precipitate a judgment in similar instances; since such mistakes tend manifestly to cherish and extend those prejudices which have been lately propagated with so much industry against a practice, in which the health and safety of the rising generation are so materially concerned.

I am, &c.

*Clements Inn, Sept.* 21, 1805. R. HALL.

